# Transgenic Targeting of *Fcrls* Creates a Highly Efficient Constitutively Active Microglia Cre Line with Differentiated Specificity

**DOI:** 10.1523/ENEURO.0549-23.2024

**Published:** 2024-07-09

**Authors:** Tobias Kaiser, Jordan Dattero, Liang Li, Mandy Chen, Minqing Jiang, Andrew Harrahill, Oleg Butovsky, Guoping Feng

**Affiliations:** ^1^ McGovern Institute for Brain Research at MIT, Cambridge, Massachusetts 02139; ^2^Departments of Brain and Cognitive Sciences, Cambridge, Massachusetts 02139; ^3^Bioengineering, Massachusetts Institute of Technology, Cambridge, Massachusetts 02139; ^4^Department of Neurology, Brigham and Women’s Hospital, Harvard Medical School, Boston, Massachusetts 02115; ^5^Evergrande Center for Immunologic Diseases, Brigham and Women’s Hospital, Harvard Medical School, Boston, Massachusetts 02115; ^6^Stanley Center for Psychiatric Research, Broad Institute of MIT and Harvard, Cambridge, Massachusetts 02142

**Keywords:** BAM, Cre, CreERT2, Fcrls-Cre, microglia, neuroimmune

## Abstract

Microglia carry out important functions as the resident macrophages of the brain. To study their role in health and disease, the research community needs tools to genetically modify them with maximum completeness in a manner that distinguishes them from closely related cell types, such as monocytes. While currently available tamoxifen-inducible CreERT2 lines can achieve the differentiation from other cells, the field needs improved and publicly available constitutively active Cre lines, especially ones with favorable efficiency and specificity profiles for studies where high recombination efficiency is imperative and where tamoxifen administration is contraindicated. Here, we leverage the microglia-specific *Fcrls* gene to generate mice expressing Cre. Using genomic methods, we show correct positioning of the transgene and intact microglia homeostasis in *Fcrls-2A-Cre* mice. Crossing *Fcrls-2A-Cre* mice to four different reporters, we demonstrate highly efficient recombination in microglia across differentially sensitive loxP alleles in different genomic contexts, indicating robust applicability of the line. Further, we show that microglia recombine a loxP reporter during early embryonic development, supporting the use of the line for developmental studies. Finally, using immunofluorescence and flow cytometry, we reveal that most border-associated macrophages are also targeted whereas only few liver and spleen macrophages and virtually no white blood cell subsets exhibit Cre activity, distinguishing this line from another publicly available Cre line, *Cx3cr1-Cre^M^*. *Fcrls-2A-Cre* mice are immediately available (JAX #036591) and serve as a valuable addition to the community's microglia toolbox by providing highly efficient constitutive Cre activity with excellent specificity, particularly for studies where tamoxifen administration is undesirable.

## Significance Statement

When selecting a Cre driver line to study microglia, investigators must weigh strengths and weaknesses of available lines to make the best choice for their application. These tradeoffs include (1) availability and ease of employment, (2) chromosomal positioning of Cre with respect to the floxed allele, (3) Cre activity and completeness of recombination across the microglia population, (4) specificity with respect to acceptable off-target cell types and tissues, (5) temporal aspects including earliest onset of Cre expression or inducibility, (6) robustness in disease contexts, and (7) potential perturbation of microglia homeostasis. Considering these tradeoffs, it is evident that there may not be a one-size-fits-all solution but an application-based preference. *Fcrls-2A-Cre* mice provide an excellent option in the microglia toolbox.

## Introduction

Microglia are the resident macrophages of the brain (Lawson et al., 1990). Functionally, their phagocytic and secretory activities play a role in neurogenesis, development of neuronal connectivity, myelin maintenance, and survival of neurons (Stevens et al., 2007; Sierra et al., 2010; Paolicelli et al., 2011; Schafer et al., 2012; Ueno et al., 2013; Hagemeyer et al., 2017; Wlodarczyk et al., 2017; Weinhard et al., 2018). In addition, microglia react to perturbations such as vascular injury, multiple sclerosis lesions, and neurodegeneration (Itagaki et al., 1989; Davalos et al., 2005; Ransohoff, 2016; Aguzzi and Zhu, 2017; Keren-Shaul et al., 2017; Mathys et al., 2017; O’Loughlin et al., 2018). Dissecting the role of microglia in these processes hinges on mouse lines that enable conditional gene manipulation in microglia. Currently, several transgenic lines are available for constitutive (Cre) or inducible (CreERT2) deletion of floxed sequences. For Cre lines, loci harnessed include *Tie2*, *Csf1r*, *Lyz2*, *Cx3cr1*, *Cx3cr1/Sall1* (Clausen et al., 1999; Kisanuki et al., 2001; Ferron and Vacher, 2005; Kim et al., 2006; Samokhvalov et al., 2007; Yona et al., 2013; Orthgiess et al., 2016), and, more recently, *Crybb1* (Brioschi et al., 2023). For CreERT2, the currently most faithful lines target *Tmem119*, *HexB*, *P2ry12*, or *Cx3cr1* (Parkhurst et al., 2013; Yona et al., 2013; Kaiser and Feng, 2019; Masuda et al., 2020; McKinsey et al., 2020). When using these lines to study microglia, there are two major considerations. First, currently available constitutively active Cre lines often recombine floxed alleles in additional major non-myeloid cell types, as well as unwanted myeloid cells, such as monocytes (Orthgiess et al., 2016; Haimon et al., 2018; Masuda et al., 2020). Second, the high-fidelity CreERT2 lines require tamoxifen administration, which is a relative contraindication for developmental studies, studies of sex-specific effects, and laboratories with limited access or facilities. Thus, there is a critical need in the field for a constitutively active Cre line that distinguishes microglia from other closely related peripheral and central myeloid cells such as blood monocytes as well as peripheral macrophages (Wieghofer and Prinz, 2016; Haimon et al., 2018).

Recently, profiling of genes enriched in microglia compared with other myeloid cells revealed several candidate genes for the generation of new Cre lines (Butovsky et al., 2014). Fc receptor- like S, scavenger receptor (*Fcrls*) is expressed across all microglia subsets based on single cell RNA sequencing studies (Hammond et al., 2019; Li et al., 2019). Adding to this, bulk RNA sequencing along development shows that *Fcrls* expression commences early during embryonic development (Mass et al., 2016). In contrast, *Fcrls* is not expressed in monocytes and other peripheral immune cell subsets based on data made available through the ImmGen database (ImmGen.org). Crucially, *Fcrls* is also not expressed in neurons (Bennett et al., 2016; Saunders et al., 2018). Together, these data make *Fcrls* a suitable target locus for the generation of a microglia-specific Cre line. Here, we report the generation and characterization of an *Fcrls-2A-Cre* knock-in mouse line, wherein microglia express Cre recombinase. By crossing this newly generated mouse line to four different floxed reporter alleles, we demonstrate functional Cre activity in all microglia and a large subset of border-associated macrophages (BAMs) in the brain. Further, we demonstrate that Cre is inactive in astroglia, oligodendroglia, and neurons and modestly but non-negligibly active in liver and spleen macrophages. Most strikingly, we found virtually no Cre expression in white blood cells, unlike the best currently available constitutively active Cre line, *Cx1cr1-Cre^M^*. Thus, this new mouse line will expand the microglia research toolbox by adding a highly efficient constitutively active Cre line with differentiated specificity. This line is immediately available to investigators from JAX (Stock #036591).

## Materials and Methods

### Animal work

All animal procedures were performed in accordance with MIT's animal care committee's regulations. Mice were bred to a C57BL/6J. For all experiments, mice of both sexes were used, and no apparent sex differences were observed. Mice were either created in-house as part of this project (*Fcrls-2A-Cre*) or obtained from external sources. *Ai14(RCL-tdT)-D* (Stock #007914), *Tie2-Cre aka B6.Cg-Tg(Tek-cre)1Ywa/J* (Stock #008863), *B6J.129(Cg)-Rpl22^tm1.1Psam^/SjJ* (Stock #029977), *B6.129X1-Gt(ROSA)26Sor^tm1(EYFP)Cos^*/J (Stock #006148), and B6J.129(B6N)-Gt(ROSA)26Sor^tm1(CAG-cas9*,-EGFP)Fezh^/J (Stock #026175**)** were obtained from JAX. *Cx3cr1-Cre^M^* was generated by IVF using C57BL/6N oocytes and sperm from MMRRC (036395-UCD).

### Generation of transgenic animals using CRISPR/Cas9

To generate *Fcrls-2A-Cre* knock-in mice, donor DNA template encoding ribosome-skipping peptide *porcine teschovirus-1 polyprotein* and *Cre* (*P2A-Cre*) was synthesized. The sequence was flanked by sequences of 1.5 kb homologous to 5′ and 3′ regions around the *Fcrls* stop codon. The template was injected into fertilized mouse oocytes together with three crRNAs (UCUAGAUCUUCAGAAAGUGC, CUAGAUCUUCAGAAAGUGCU, AGACCUCCUACUUUCUGCAC, Synthego) that cut sequences flanking the stop codon.

The *Fcrls-2A-Cre* knock-in donor DNA template was generated by synthesizing the left homology arm (LHA), Cre, and right homology arm (RHA) fragments using PCR (Phusion polymerase). Using PCR with genomic DNA template, the left homology arm was created with forward and reverse primers (5′- gcggccgcacgcgtttaattaagtgtacccaaagtgcgtttggtg-3′ and 5′- acgtctccagcctgcttcagcaggctgaagttagtagcgaaagtgctgggtaagactgtg-3′). The right homology arm was created from genomic DNA with forward and reverse primers (5′-agatctagagaccagagaccatc-3′ and 5′-agaggttgattatcgataagcttgatatcggacgggaagatgagtgaacttac-3′). The Cre insert was created from PL450-IRES-Cre using forward and reverse primers (5′- gctgaagcaggctggagacgtggaggagaaccctggacctatggccaatttactgaccgt-3′ and 5′- atggtctctggtctctagatctctaatcgccatcttccagca-3′).

A generic pAAV destination plasmid was digested with XbaI and EcoRI (New England Biolabs), and the three fragments (LHA, P2A-Cre, RHA) were inserted by Gibson cloning (HiFi Assembly Mix, New England Biolabs) using the four-fragment protocol. Positive clones were isolated, DNA was prepared, and the PAM site of the 3′most sgRNA was mutated using site-directed mutagenesis. Resulting plasmids were purified and sequenced.

For zygote injection, CRISPR/Cas RNP mixtures were prepared freshly on the morning of the day of injection. Briefly, water to a final volume of 100 µl was mixed with final concentrations of 10 mM TrisHcl buffer, 0.61 µM of each of the three crRNA (1.83 µM total), 2.44 µM tracrRNA (sequence proprietary to Synthego) and heated to 95°C for 5 min. Heated mixtures were cooled to room temperature for 10 min and 30 ng/µl EnGen Cas9 protein (New England Biolabs) was added. Mixtures were incubated at 37°C for 15 min to form Cas9 to crRNA-tracrRNA complexes. Final concentrations of 10 ng/µl donor DNA and 10 ng/µl recombinant RAD51 protein were added. Mixtures were kept on ice until use, when they were incubated at 37°C for 15 min followed by centrifugation at 10.000 rpm for 1 min to prevent clogging of the micropipette.

Injection of mixtures was carried out using standardized protocols of the transgenics facility in zygotes obtained from pure C57BL/B6J mice (JAX). Zygotes were implanted into surrogate females and carried to term.

### Genetic analysis of *Fcrls-2A-Cre* mice

F0 founder mice and F1 mice were genetically examined by amplifying sequences spanning the 5′ and 3′ junction and including the entire inserted transgene. Specifically, high-quality DNA was obtained from ear punches using a tissue DNA extraction kit according to the manufacturer’s instructions (Macherey-Nagel). Amplicons spanning these sequences were generated using Phusion polymerase using primers for a 5′ fragment (ggagctgcttaagagtatgcac, ggcaaattttggtgtacggtc) and a 3′ fragment (gtcatgaactatatccgtaacctgg, tctggtagtacatcatactgattaagac) that are unique to the knock-in allele. PCR products were purified and Sanger sequenced for positive founders. For genotyping of mice from F1 and later generations, a simplified protocol was employed using three primers (agacgatttgggatggtttg, tggctggaccaatgtaaatattg, acagctgaagtctggaagtc) that yield a wild-type band of 284 bp and a Cre band of 194 bp.

### Preparation of cell suspensions

Microglia and blood monocytes for single cell suspensions for flow cytometry were prepared as follows. Mice were euthanized with an isoflurane overdose and promptly transcardially perfused with ice-cold HBSS. During perfusion, for adult mice, 2 ml of whole blood was collected from the right atrium into tubes containing 40 µl of 10% (w/v) EDTA. The 2 ml of whole blood was added to 40 ml of red blood cell lysis buffer (Abcam, ab204733) and incubated for 10 min at room temperature to lyse red blood cells. The suspension containing lysed RBCs and white blood cells was spun down at 300 × *g* for 5 min at 4°C, resuspended in 10 ml of ice-cold HBSS, and pelleted again at 300 × *g* for 5 min at 4°C. WBCs were resuspended in 500 µl of ice-cold FACS buffer (HBSS, 0.5% BSA, 1 mM EDTA) and subject to staining. In parallel with the WBC enrichment, brains were rapidly dissected into 2 ml of ice-cold HBSS, and the cerebella and brainstem were removed. Brains were minced into small pieces and transferred to a dounce homogenizer containing 5 ml of ice-cold HBSS with 20 µg/ml DNase I (Worthington, DPRF, LS006343). Tissue chunks were homogenized with seven loose and 15 tight strokes (for adult mice: 15 loose and 15 tight strokes), and the homogenate was transferred to a 50 ml of falcon tube through a prewet 70 µm strainer. The strainer was rinsed with HBSS to top off the volume of each sample to 10 ml. Filtered homogenates were transferred to 15 ml of falcon tubes and spun at 300 × *g* for 5 min at 4°C. For adult mice, supernatants were carefully removed and pellets resuspended in 10 ml of ice-cold 40% Percoll in HBSS. Samples were spun at 500 × *g* for 30 min at 4°C with full acceleration and deceleration. Myelin and debris from the supernatant were carefully removed and pellets resuspended in 10 ml of ice-cold HBSS. Following another spin at 300 × *g* for 5 min at 4°C, the supernatant was removed and the microglial pellet resuspended in 1 ml of FACS buffer (0.5% BSA HBSS, 1 mM EDTA). For neonatal mice, Percoll centrifugation was not performed and washed cell pellets were used directly.

### Staining and flow cytometry

Cell suspensions were transferred to 2 ml of Eppendorf microcentrifuge tubes, and a small fraction of sample was removed for single-color controls. To stain dead cells, live/dead violet (1:500 to 1:1,000, Thermo Fisher Scientific, L34955) or DAPI (1:1,000) was added and incubated for 5 min on ice. Tubes with live/dead-stained cells were topped off to 2 ml with FACS buffer (0.5% BSA HBSS, 1 mM EDTA) and spun down at 300 × *g* for 5 min at 4°C. Pellets were resuspended and incubated with 1:200 Mouse Fc Block (BD, 2.4G2, 553142) on ice for 15 min. Samples were incubated with 1:200 rat anti-CD45 (BioLegend, 30-F11, different conjugates) and rat anti-Cd11b (BioLegend, M1/70, different conjugates), CD206 (141711, BioLegend), Ly6G (561105, BD), Ly6C (AL-21, BD Biosciences) at 4°C. Tubes were topped off to 2 ml with ice-cold FACS buffer and microglia pelleted at 300 × *g* for 5 min at 4°C. Supernatants were removed and microglia resuspended in 500 µl. Resuspended microglia were filtered through corning strainer polystyrene tubes (Corning, 352235). Flow cytometry data was acquired on Aria II, Fortessa HTS, LSRII HTS, and Sony Symphony and analyzed using FlowJo.

For white blood cell analysis in *Fcrls-2A-Cre* mice, cells isolated as described above and stained with primary antibodies. Antibodies were chosen as previously reported (Masuda et al., 2020) and directed against CD11b (M1/70, BioLegend), CD45 (30-F11, Thermo Fisher Scientific), Ly6C (AL-21, BD Biosciences), Ly6G (1A8, BD Biosciences), CD115 (AFS98, Thermo Fisher Scientific), CD11c (N418, Thermo Fisher Scientific), MHC class II (M5/114.15.2, Thermo Fisher Scientific), CD3e (eBio500A2, Thermo Fisher Scientific) for 45 min at 4°C. After washing, cells were washed and resuspended in buffer containing 3% BSA.

### RNA isolation and quantitative PCR (qPCR)

For RNA isolation from microglia, the RNeasy Micro kit (Qiagen) was used, and 50,000 cells were sorted directly into 350 µl of RLT Plus buffer containing 143 mM 2-mercaptoethanol. RNA was isolated using kit according to the manufacturers specifications resulting in 12–13 µl of RNA at 1–2 ng/µl. RNA was reverse transcribed using the iScript Advanced low input kit (Bio-Rad). qPCR was run with 0.25 µM of each primer and cDNA as input using the SsOAdvanced Universal SYBR Green Supermix (Bio-Rad) on a CFX96 real-time system. The following primers were used (5′ to 3′). Fcrls-F TGTGCTTGCTGCTTCTGGTC, Fcrls-R CCTGTGCAGCTTATAACTACTTGGTC, P2ry12-F CGCACGGACACTTTCCCGTAT, P2ry12-R GGAACTTGCAGACTGGCATCT, Tmem119-F CCTTCACCCAGAGCTGGTTC, Tmem119-R GGCTACATCCTCCAGGAAGG, 18S-F ACGGAAGGGCACCACCAGGA, 18S-R CACCACCACCCACGGAATCG, Actb-F CTAAGGCCAACCGTGAAAAG, Actb-R ACCAGAGGCATACAGGGACA. Differential gene expression analysis was performed using built-in software for the Bio-Rad CFX96 real-time system.

### Immunofluorescence staining and imaging

Adult Ai14 or Rpl22-HA reporter-harboring mice were deeply anesthetized and perfused with 25 ml of phosphate buffered saline (PBS) followed by 25 ml of 4% paraformaldehyde (PFA) in PBS. R26YFP reporter-harboring mice were euthanized with isoflurane overdose without perfusion. Brains, livers, and spleens were surgically removed and postfixed in the fixative at 4°C for 24 h. Fixed brains, livers, and spleens were washed in PBS once and sliced into 100-µm-thick sagittal slices using a Leica VT1000S. For immunostaining of HA in Rpl22-HA mice, mice were perfused with HBSS and brains immersion fixed in 2% PFA at room temperature for 4 h. Brains were washed and sectioned on a vibratome at 100 µm. Slices were washed twice in PBS, permeabilized in 1.2% Triton X-100 in PBS for 15 min, washed twice in PBS, and subject to incubation in blocking solution (5% normal goat serum, 2% bovine serum albumin, 0.2% Triton X-100 in PBS; or only 2% BSA when a goat primary antibody was used). Blocked sections were incubated with primary antibodies for IBA1 (1:500, Synaptic Systems, 234006), HA (1:1,000, C29F4, Cell Signaling Technology, used with lighter fixation, see below), Collagen IV (1:500, Millipore, Ab769), GFP (1:1,000, Invitrogen, A11122, 1:500, Aves Labs, GFP-1020), S100B (1:1,000, HPA015768, Sigma), Olig2 (1:1,000, Millipore, AB9610), NeuN (1:1,000, Millipore, MAB377), Lectin-647 (1:200, DL-1178-1, VectorLabs), or CD163 (1:200, Abcam, ab182422, antigen retrieval required) for 24 h at 4°C. Primary antibody incubation was followed by three washes in PBS and incubation with species-matched and Alexa fluorophore-conjugated secondary antibodies raised in goat or donkey (Invitrogen, 1:1,000) for 2 h. DAPI (1:10,000) was included in a washing step or secondary antibody incubation. Slices were washed three times in PBS and mounted and coverslipped using vectashield H-1000 mounting medium.

For immunostaining of CD163, an antigen retrieval step was included. Briefly, vibratome slices were washed twice in PBS, incubated in a retrieval buffer (10 mM sodium citrate, pH 8.5) for 5 min, followed by incubation in the retrieval buffer at 80°C for 30 min. Sections were cooled to room temperature and washed twice in PBS. Blocking and immunostaining were then carried out as described above.

For imaging, slides were scanned on an Olympus Fluoview FV1000 and FV3000 fixed stage confocal microscope (high power magnifications) using built-in software. For coexpression analysis, single plane 10× or 20× high-power fields were analyzed.

For studies of protein expression in embryonic brain, timed matings were set up by pairing *Fcrls-2A-Cre* male mice with Ai14 female mice. Males were removed the morning after the mating was set up, which was considered Day 0.5. Females were examined for pregnancy 12 d later and pregnant Ai14 females were euthanized. Uteri and embryos were dissected in ice-cold HBSS, and embryos were fixed by immersion in 4% PFA overnight. Embryos were embedded in 2% Agarose and sliced at 100 µm using a Leica VT1000S. Slices were washed twice in PBS and stained against IBA1 as described above.

### Data analysis

Micrographs from confocal imaging were quantified in FIJI by counting immunostaining-positive regions of interest. Flow cytometry data was gated and processed using FlowJo. qPCR data were analyzed using the built-in software suite of the Bio-Rad CFX96 real-time system. Quantitative data from the qPCR, flow cytometry, and immunofluorescence were analyzed using GraphPad Prism. The number of biological replicates and statistical testing is indicated in the figure caption for each experiment.

## Results

### Generation of a constitutively active microglia Cre line (*Fcrls-2A-Cre*) using a CRISPR/Cas9-mediated knock-in strategy

**Figure 1.**
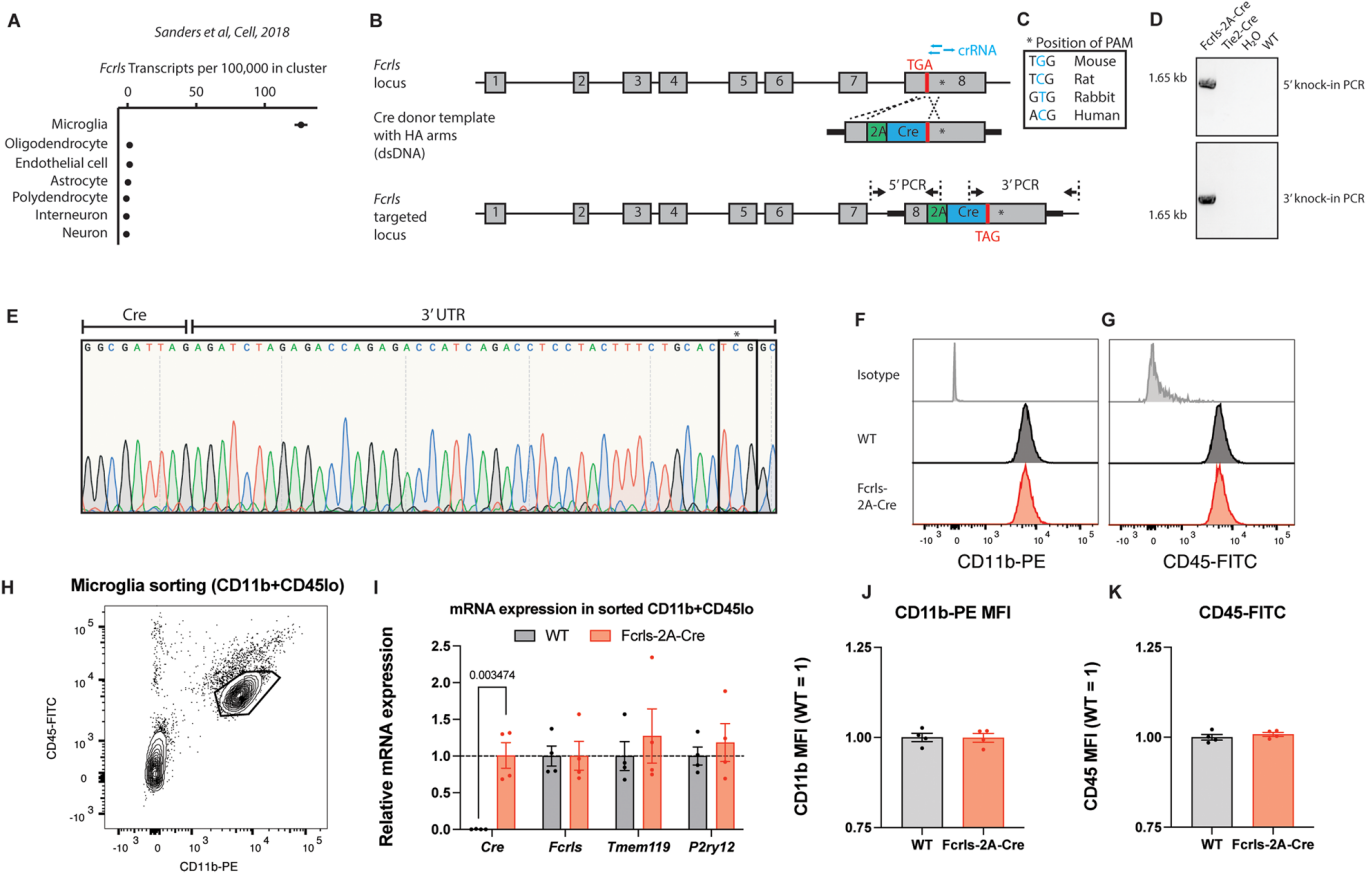
Generation of a constitutively active microglia Cre line through targeting of the *Fcrls* locus using CRISPR/Cas9. ***A***, *Fcrls* expression across cell subsets in frontal cortex single-cell RNA sequencing data from Saunders et al. (2018). ***B***, Schematic representation of murine *Fcrls* locus and knock-in approach to insert a *2A-Cre* cassette into the stop codon of *Fcrls* in exon 8 (not drawn to scale). Three crRNAs (blue bars) were selected to introduce double strand breaks at the stop codon and in the 3′ UTR. PCR primers to check for on-target insertion are indicated as arrows. ***C***, The 3′ UTR-targeting sgRNA was selected such that nucleotide substitution required for silencing of the NGG PAM for CRISPR/Cas-mediated knock-in alters a nonconserved nucleotide (inset box). ***D***, Representative agarose gel electrophoresis image of the indicated 5′ and 3′ PCR amplicons spanning the junctions of inserted *2A-Cre* transgene and target locus in founder animals. ***E***, Sanger sequencing chromatogram of 3′ amplicon showing the G→C mutation in the founder animals. ***F***, Representative flow cytometry density plot indicating gating of microglia for isolation (CD11b + CD45lo) in postnatal day 8 animals. ***G***, RT-qPCR for *Cre* and *Fcrls* mRNA and two microglia homeostasis genes from sorted microglia. *N* = 4 mice. Multiple *t* tests with Benjamini, Krieger, and Yekutieli correction for multiple testing. *q* = 0.003 for *Cre*, others are not significant. ***H–I***, Histogram plot for CD11b and CD45 expression on P8 microglia as gated in panel ***E***. ***J–K***, Quantification of CD11b and CD45 protein expression on P8 microglia. *N* = 4 mice. Unpaired *t* test. Not significant.

Cre-driver lines and conditional alleles are critical in the quest to understand the roles of microglia in health and disease. Several lines utilizing Cre or its inducible form CreERT2 have been previously generated and are now widely used in research (excellently reviewed by Wieghofer and Prinz, 2016; Bennett and Bennett, 2020; Miron and Priller, 2020; Dumas et al., 2021). While these lines have collectively enabled the field to make significant headway in understanding microglia biology, additional publicly available transgenic mouse lines would provide valuable additions to our mouse line repertoire if they efficiently targeted microglia while obviating the need for tamoxifen-mediated Cre induction and while avoiding targeting of monocytes. Among a group of recently discovered microglia-enriched genes, we initially chose *Tmem119* to generate a constitutive *Tmem119-2A-Cre* mouse line in addition to the previously published *Tmem119-2A-EGFP* and *Tmem119-2A-CreERT2* lines (unpublished data, Bennett et al., 2016; Kaiser and Feng, 2019). Unlike the EGFP and CreERT2 lines which showed specificity for microglia, the constitute Cre mouse line displayed widespread Cre activity in brain endothelial cells and was discontinued (unpublished data). Further mining of public datasets suggested that microglia-enriched *Fcrls* is a suitable locus for Cre knock-in due to its high expression across all microglia subsets, early onset of expression, and complete absence of expression in monocytes (Butovsky et al., 2014; Hammond et al., 2019; Li et al., 2019, ImmGen.org). Further, among major cell types in the CNS including oligodendrocytes, astrocytes, endothelial cells, and neurons, *Fcrls* is only expressed in microglia (Fig. 1*A*). To generate *Fcrls-2A-Cre* mice, we created a double-strand DNA targeting vector containing *2A-Cre*, 1.5 kb homology arms, and an additional PAM (protospacer adjacent motif) point mutation and injected mouse zygotes with a mixture of this targeting vector, Cas9 protein, and three crRNAs (Fig. 1*B*,*C*). This design inserts *2A-Cre-STOP* into the STOP codon of *Fcrls*, resulting in a single transcript that encodes both *Fcrls* and *Cre* by means of ribosomal skipping. Implantation of the zygotes into surrogates resulted in 14 live births (F0 founders) which we subsequently screened and genotyped with primer pairs having binding sites outside the homology arms and inside the transgene to generate amplicon uniquely present in mice with correct knock-in (Fig. 1*B*). We found four out of the 14 founders positive for the insert in the *Fcrls* locus and crossed them to C57BL/6J to generate F1 animals, which we also found to harbor the insert in the *Fcrls* locus (Fig. 1*D*). Using Sanger sequencing, we verified intactness of the genomic sequence and the 3′UTR single-base change introduced to create the PAM mutation that prevented recutting by CRISPR/Cas (Fig. 1*E*). Together, these data demonstrate that we successfully generated *Fcrls-2A-Cre* mice.

A drawback of using Cre to recombine floxed genes of interest and measure associated phenotypes is that the genomic Cre insertion or the Cre protein itself can affect cellular function. Specifically for microglia, Gosh and coworkers recently demonstrated that high Cre expression in *Cx3cr1-CreERT2* mice disrupts microglia homeostasis during early postnatal development (Sahasrabuddhe and Ghosh, 2022). To test if *Fcrls-2A-Cre* mice display similar abnormalities, we analyzed microglia from postnatal day 8 (P8) mice by flow cytometry and sorted them for subsequent RT-qPCR of homeostatic genes (Fig. 1*F*). Unlike *Cx3cr1-CreERT2* P8 microglia analyzed by Sahasrabuddhe and Ghosh that displayed elevated CD11b and CD45 expression as well as disrupted homeostasis, we found microglia isolated from *Fcrls-2A-Cre* mice to be indistinguishable from WT microglia (Fig. 1*G–K*). Together, these data suggest that the microglia state, at least based on these studies limited in scope, is unperturbed in *Fcrls-2A-Cre* mice.

### *Fcrls-2A-Cre* mice effectively recombine floxed DNA in all microglia and most BAMs

**Figure 2.**
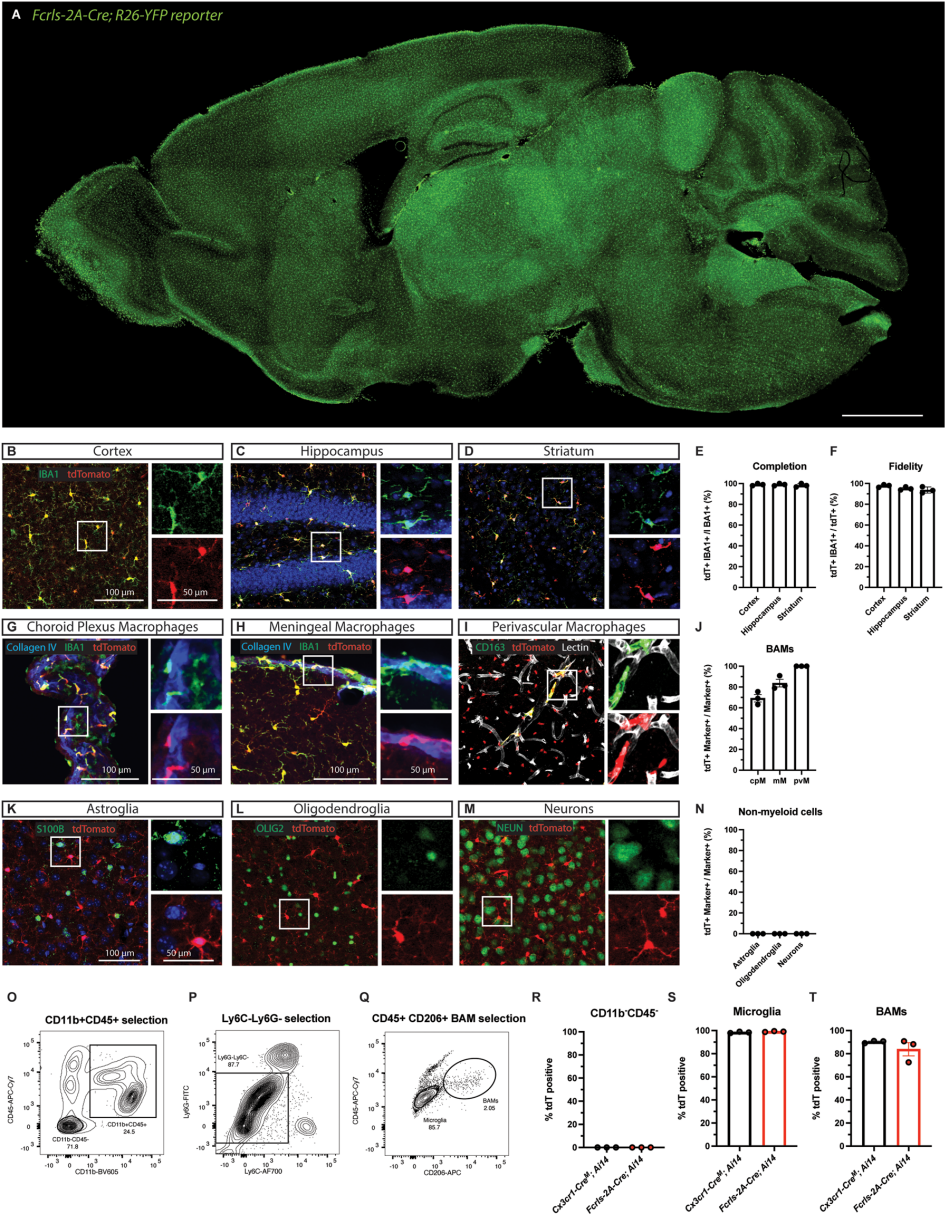
*Fcrls-A-Cre* mice effectively recombine floxed DNA in all microglia and most BAMs. ***A***, Representative epifluorescence micrograph of immersion fixed brain of adult (2 months of age) *Fcrls-2A-Cre^+/^*^−^*;R26YFP^+/^*^−^ reporter mice. Scale bar 1 mm. ***B–D***, Representative confocal micrographs of immunostained slices from adult (1–4 months of age) *Fcrls-2A-Cre^+/^*^−^*;Ai14^+/v^* mice showing tdTomato due to Cre recombination (red) and microglia (green) in cortex, hippocampus, and striatum. ***E***, ***F***, Quantification of the completion and fidelity of Cre activity in IBA1^+^ parenchymal macrophages in the brain. *N* = 3 mice. ***G–I***, Representative confocal micrographs of different BAM subsets, including choroid plexus macrophages (green, anatomical, Collagen IV-adjacent), meningeal macrophages (green, Collagen IV-adjacent), and perivascular macrophages (green, CD163^+^, Lectin-adjacent). ***J***, Quantification of *Fcrls-2A-Cre*-mediated recombination in BAM subsets. *N* = 3 mice. ***K–M***, Representative confocal micrographs of immunostaining for S100B (green, astroglia), OLIG2 (green, oligodendroglia), and NEUN (green, neurons) in slices from *Fcrls-2A-Cre^+/^*^−^*;Ai14^+/^*^−^ mice. ***N***, Quantification of *Fcrls-2A-Cre*-mediated recombination in non-myeloid brain cells (astroglia, oligodendroglia, neurons). *N* = 3 mice. ***O–Q***, Representative flow cytometry density plots showing gating of microglia (CD11b^+^CD45^lo^Ly6C^−^Ly6G^-^CD206−) and BAMs (CD11b^+^CD45^+^Ly6C^−^Ly6G^−^CD206^+^). ***R–T***, Quantification of *Fcrls-2A-Cre*- or *Cx3cr1-Cre^M^*-mediated (as positive control) recombination in CD11b^−^CD45^−^ non-myeloid cells, microglia, and BAMs. *N* = 3 mice per group.

To investigate the activity of Cre recombinase, we crossed *Fcrls-2A-Cre* mice to R26-YFP and Ai14 tdTomato mice, which report recombination with tdTomato fluorescence (Madisen et al., 2010). Examining brain slices from the *Fcrls-2A-Cre; R26YFP* mice, it was readily apparent that the reporter was expressed throughout the brain in cells resembling morphology and tile-like distribution of microglia (Fig. 2*A*). Further, neither larger neuronal projections nor prominent Purkinje neurons in the cerebellum expressed the reporter. We prepared *Fcrls-2A-Cre^+/^*^−^*; Ai14^+/^*^−^ mice (short *Fcrls-2A-Cre; Ai14*, as only heterozygous mice were used throughout the study) for immunofluorescence staining of several regions. High power confocal micrographs of the cortex, hippocampus, and striatum showed that parenchymal IBA1-positive microglia expressed tdTomato (Fig. 2*B–D*). All other CNS regions including the spinal cord showed robust tdTomato expression (data not shown). We quantified the confocal micrographs for completion (fraction of IBA1^+^ parenchymal microglia expressing tdTomato) and fidelity (fraction of tdTomato^+^ cells being parenchymal IBA1^+^ microglia) and found that parenchymal microglia were completely labeled and all labeled cells in the parenchyma were microglia (Fig. 2*E*,*F*). In addition to parenchymal microglia, we examined BAMs, as these cells are closely related to microglia and at least a subset of them is known to express *Fcrls* in single-cell RNA-sequencing analyses (Hammond et al., 2019; Li et al., 2019). Confocal micrographs of coimmunostained brain sections leveraging location of the cells showed that choroid plexus macrophages, meningeal macrophages, and perivascular macrophages all harbor recombined alleles to varying degrees, indicating Cre activity in a substantial fraction of these cells (Fig. 2*G–J*). To further examine the specificity and examine expression in major subsets of non-myeloid cell types, we stained brain slices against S100B (astroglia), OLIG2 (oligodendrocyte lineage cells), and NEUN (neurons). Confocal micrographs revealed that none of the subsets expressed tdTomato (Fig. 2*K–N*).

Complementing the histological analysis, we employed flow cytometry on dissociated brain tissue to test the recombination activity of *Fcrls-2A-Cre* mice with an orthogonal approach. Specifically, we probed tdTomato expression in several populations, including non-myeloid cells, microglia, and BAMs that we gated based on reported surface markers (Fig. 2*O–Q*). While none of the non-myeloid cells were tdTomato-positive, we observed near complete tdTomato-positivity among microglia and BAMs in *Fcrls-2A-Cre; Ai14* and *Cx3cr1-Cre^M^; Ai14* mice which we used as a positive control (Fig. 2*R–T*). These findings collectively demonstrate that *Fcrls-2A-Cre* mice recombine floxed DNA sequences with high efficiency in all microglia and most BAMs.

### *Fcrls-2A-Cre* mice efficiently recombine floxed DNA in microglia across differentially sensitive Cre-loxP reporter strains and in early development

**Figure 3.**
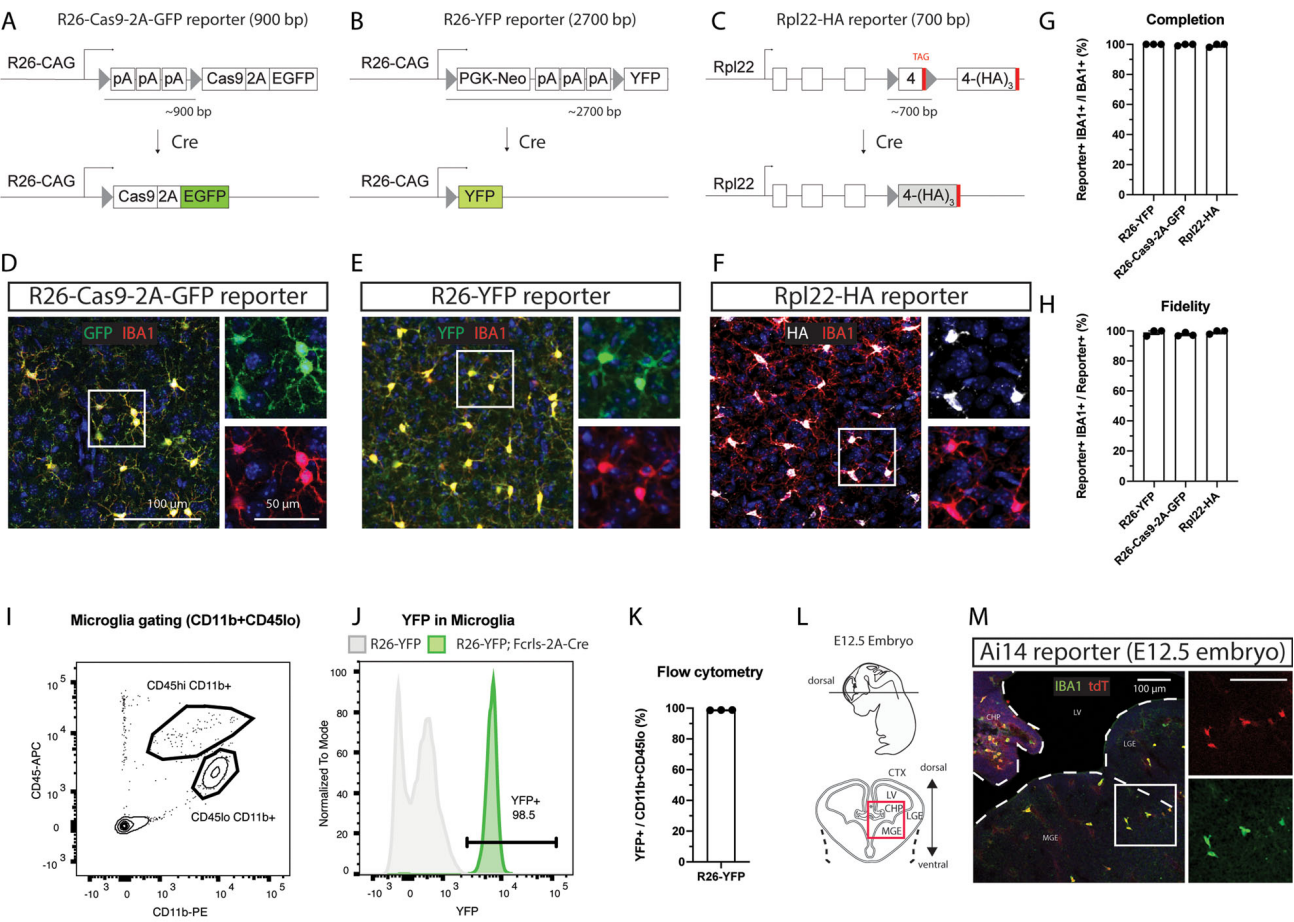
*Fcrls-2A-Cre* mice efficiently recombine floxed DNA in microglia across differentially sensitive Cre-loxP reporter strains and in early development. ***A–C***, Schematic representation of different Cre-loxP reporter stains employed. The different strains utilize different lox-stop reporter strategies and differ in the spacing between loxP sites, as well as the genomic context. Cre-mediated recombination of loxP sites results in expression of fluorescent reporters (EGFP, YFP) or an immunostainable HA-tagged ribosomal subunit (Rpl22-3xHA). ***D–F***, Representative confocal micrographs of cortex in immunostained slices from *Fcrls-2A-Cre^+/^*^−^*;Reporter^+/^*^−^ mice showing GFP (green), YFP (green), or HA (white) detection possible due to Cre recombination in IBA1-immunostained microglia (red). ***G–H***, Quantification of the completion and fidelity of Cre activity in IBA1^+^ parenchymal macrophages in the brain of the different reporter strains. *N* = 3 mice per group. ***I***, ***J***, Representative flow cytometry density plot for microglia gating and histogram showing YFP expression in microglia. ***K***, Quantification of YFP expression in microglia flow cytometry. *N* = 3 mice. ***L***, Schematic representation of E12.5 embryo, cryo-sectioning plane, and imaging field of view (red box). CTX, cortex; CHP, choroid plexus; LGE, lateral ganglionic eminence; LV, lateral ventricle; MGE, medium ganglionic eminence. ***M***, Representative confocal micrograph of immunostained slices from *Fcrls-2A-Cre^+/^*^−^*;Ai14^+/^*^−^ mice showing Cre-dependent expression of tdTomato (red) and IBA1-positive microglia (green). Representative for *N* = 2 E12.5 embryos.

The efficiency of Cre-mediated loxP site recombination and thus the completeness and fidelity depend on the expression levels of Cre and the distance between loxP sites among other factors (Glaser et al., 2005; Stifter and Greter, 2020). Previous carefully designed work using inducible microglia CreERT2 lines showed varying degrees of leakiness in the context of sensitive reporters and high CreERT2 expression levels (Chappell-Maor et al., 2020; Van Hove et al., 2020). Conversely, inefficient recombination has been observed in the context of less sensitive reporters and lower Cre expression levels (Bedolla et al., 2023; Faust et al., 2023). To test the generalizability of loxP recombination by the *Fcrls-2A-Cre* line, we crossed *Fcrls-2A-Cre* mice to three different reporters of differential sensitivity and genomic context (Fig. 3*A–C*). Among these reporters, *R26-Cas9-2A-GFP* and *R26-YFP* were created through insertion into the transcriptionally highly active *Rosa26* locus and driven by the strong *CAG* promoter, whereas *Rpl22-HA* is driven by its endogenous promoter and integrated in the endogenous *Rpl22* locus. Further, likely due to the long distance between the loxP sites, the *R26-YFP* reporter is known to be less sensitive compared with Ai14-type reporters and a good proxy for harder-to-recombine alleles. We co-immunostained brain slices and observed reporter activity in parenchymal IBA1-expressing microglia in the cortex (Fig. 3*D–F*). We found this activity in nearly all microglia (99%) and nearly all reporter signal (97–99%) was attributable to IBA1-expressing cells (Fig. 3*G–H*). To further evaluate the completeness of recombination with an orthogonal flow cytometry assay, we dissociated and analyzed brains of *Fcrls-2A-Cre;R26-YFP* animals and noted that nearly all CD11b^+^CD45^lo^ microglia (98.7%) expressed YFP (Fig. 3*I–K*). Together, these data indicate a generalizable and high level of completeness of recombination in *Fcrls-2A-Cre* mice independent of the reporter allele used.

Microglia-specific genes are not expressed uniformly and some genes express at lower levels or in select microglia subpopulations, especially during development (Hammond et al., 2019; Li et al., 2019), which could affect the utility of the *Fcrls-2A-Cre* line for studies requiring loxP recombination. To test recombination capability at embryonic development, we immunostained brain slices prepared from E12.5 *Fcrls-2A-Cre;Ai14* embryos (Fig. 3*L*) and observed reporter activity in IBA1-expressing microglia, indicating that *Fcrls-2A-Cre* recombines floxed alleles at this early stage and is thus suitable for studies requiring early loxP recombination.

### *Fcrls-2A-Cre* mice recombine floxed DNA in a subset of peripheral macrophages while completely sparing white blood cells

**Figure 4.**
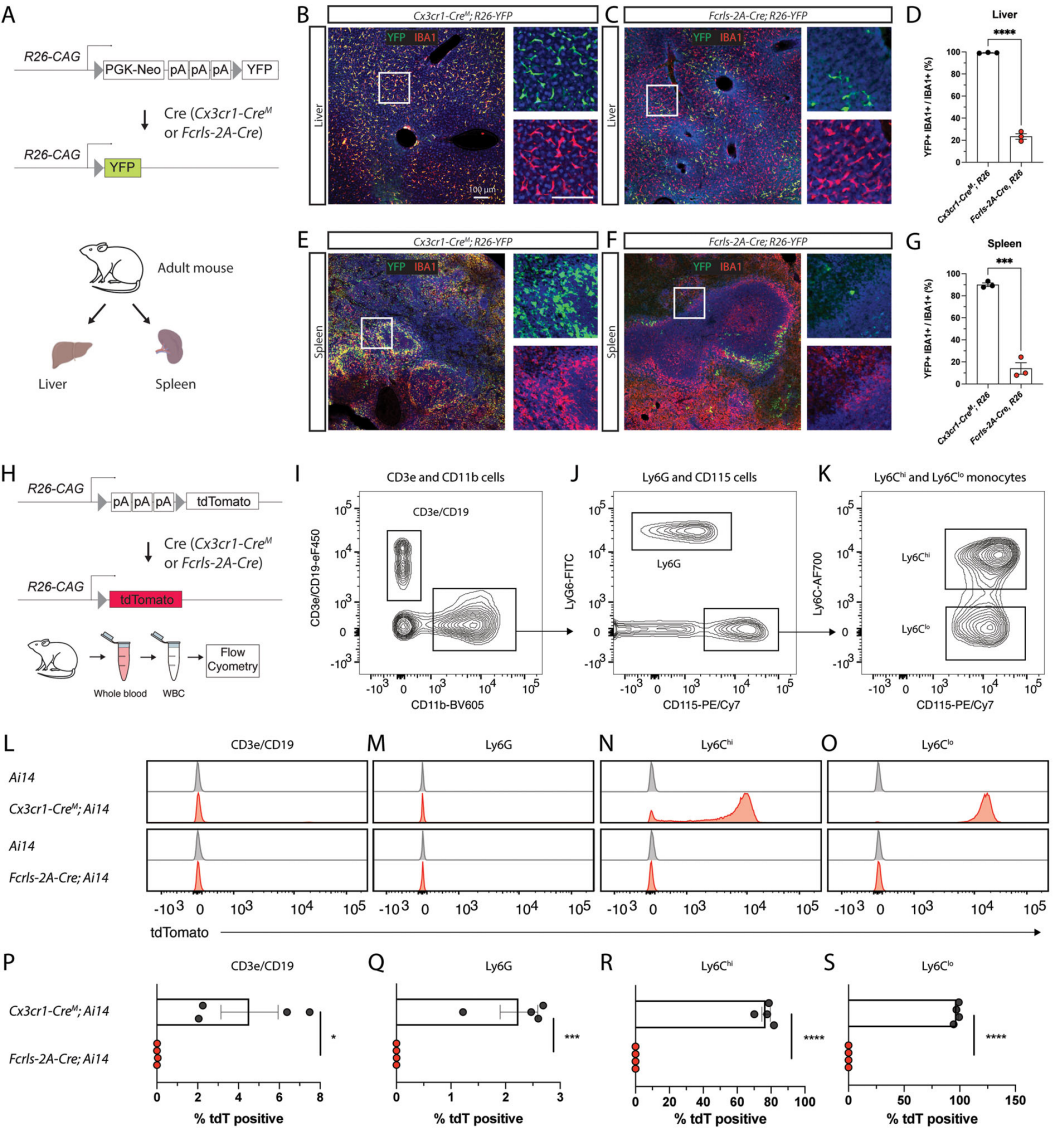
*Fcrls-2A-Cre* mice recombine floxed DNA in a subset of peripheral macrophages while completely sparing white blood cells. ***A***, Schematic representation of recombination of R26-YFP reporter in crossings with *Fcrls-2A-Cre* and *Cx3cr1-Cre^M^* (positive control) mice and organs harvested for immunostaining. ***B–C***, Representative confocal micrographs of YFP fluorescence (green) and IBA1-immunostaining (red, macrophages) in the liver of adult animals. ***D***, Quantification of YFP-positive macrophages in the liver. *N* = 3 per group. Unpaired *t* test. ***p < 0.0001. ***E***, ***F***, Representative confocal micrographs of YFP fluorescence (green) and IBA1-immunostaining (red, macrophages) in the spleen of adult animals. ***G***, Quantification of YFP-positive macrophages in the spleen. *N* = 3 per group. Unpaired *t* test. ***p < 0.0002. ***H***, Schematic representation of recombination of Ai14 reporter in crossings with *Fcrls-2A-Cre* and *Cx3cr1-Cre* (positive control) mice and white blood cell harvesting for flow cytometry. ***I–K***, Representative flow cytometry density plots showing gating for lymphocytes (CD11b-CD19 + CD3e+), granulocytes (CD11b + Ly6G+), and monocytes (CD115^+^Ly6C^hi^ and CD115^+^Ly6C^lo^). Pregated on single, live, CD45^+^ cells. ***L–O***, Histograms showing tdTomato fluorescence among the different white blood cell subset populations for *Ai14* controls, *Cx3cr1-Cre^M^; Ai14* mice, and *Fcrls-Cre; Ai14* mice. ***P–S***, Scatter plots showing the percentage of tdTomato-positive cells among all cells within a given subset in *Cx3cr1^M^-Cre^+/^*^−^*;Ai14^+/^*^−^ mice, and *Fcrls-2A-Cre^+/^*^−^*;Ai14^+/^*^−^ mice. *N* = 4 mice per group. Unpaired *t* test. *p = 0.0178, ***p = 0.0006, ****p < 0.0001.

*Fcrls* is highly enriched in microglia compared with other myeloid cells (Butovsky et al., 2014), but even minute amounts of Cre can lead to all-or-none recombination events of floxed genomic sequences. To probe whether peripheral macrophages display Cre activity in *Fcrls-2A-Cre* mice, we examined liver and spleen tissue slices from *Fcrls-2A-Cre;R26-YFP* mice along with *Cx3cr1-Cre^M^;R26-YFP* mice as a positive control and comparator (Fig. 4*A*). Across both organs, we observed near complete reporter activity (99% liver and 90% spleen) in tissues collected from *Cx3cr1-Cre^M^;R26-YFP* mice (Fig. 4*B–G*). In contrast, samples from *Fcrls-2A-Cre;R26-YFP* mice showed substantially lower, albeit nonzero, recombination in these organs (23% liver and 14% spleen, Fig. 4*B–G*), indicating a more modest targeting of peripheral macrophages, or perhaps only subsets thereof, in the *Fcrls-2A-Cre* line compared with the *Cx3cr1-Cre^M^* line.

Monocytes infiltrate the brain and mediate pathological events in conditions such as Stroke, Alzheimer’s disease, and Multiple Sclerosis (Henderson et al., 2009; Ritzel et al., 2015; Shang et al., 2016). Recently developed CreERT2 lines including *Tmem119-CreERT2* and *P2Ry12-CreERT2* almost completely avoid recombination of floxed alleles in monocytes (Kaiser and Feng, 2019; McKinsey et al., 2020). For constitutively active Cre lines, however, the currently best publicly available line, *Cx3cr1-Cre^M^* (MMRRC, Gong et al., 2010; Zhao et al., 2019), is expected to express Cre in monocytes and perhaps other white blood cells based on the endogenous expression profile of *Cx3cr1* (ImmGen.org). This inability to discern the two cell types precludes clear delineation of the role of microglia when using this line to study these disorders. To determine whether *Fcrls-2A-Cre* displays Cre activity in leukocyte subsets, we harvested white blood cells from adult *Ai14* control mice, *Fcrls-2A-Cre; Ai14* mice or *Cx3cr1-2A-Cre^M^; Ai14* mice and stained for known markers (Fig. 4*H*). Using flow cytometry, we gated single, live, CD45-expressing cells and further parsed them into CD3e/CD19^+^ lymphocytes, CD11b^+^Ly6G^+^ granulocytes, and CD115^+^Ly6C^hi^ and CD115^+^Ly6C^lo^ monocytes (Fig. 4*I–K*). Histograms of tdTomato expression revealed that while different leukocyte subsets in *Cx3cr1-Cre^M^; Ai14* mice express tdTomato, virtually no leukocyte subsets from *Fcrls-2A-Cre; Ai14* mice do (Fig. 4*L–O*). Specifically, quantification of tdTomato-positivity showed that none of the CD3e/CD19^+^ lymphocytes, CD11b^+^Ly6G^+^ granulocytes, CD115^+^Ly6C^hi^ monocytes, or CD115^+^Ly6C^lo^ monocytes expressed tdTomato in *Fcrls-2A-Cre;Ai14* mice compared with 4.5, 2.2, 77, and 97% of these cells in *Cx3cr1-Cre^M^; Ai14* mice, respectively (Fig. 4*P–S*). Together, these data demonstrate that, unlike the *Cx3cr1-Cre^M^* line, the *Fcrls-2A-Cre* line avoids undesirable Cre recombinase activity in white blood cells, especially monocytes.

## Discussion

Transgenic mouse lines enabling the manipulation of floxed target genes in microglia are critical to advance our understanding of their biology. Additional publicly available, constitutively active Cre lines that spare white blood cells would be a great addition to our repertoire of mouse lines. In this study, we addressed this need by creating an *Fcrls-2A-Cre* mouse line. Using four different reporter strains, we demonstrate that the *Fcrls-2A-Cre* line effectively and specifically recombines floxed alleles in all microglia and most BAMs. Further we show that recombination occurs early in development and that a minority of tissue-resident macrophages in some peripheral tissues exhibit Cre activity as well. Finally, utilizing flow cytometric analysis, we demonstrate that Cre is virtually inactive in different white-blood cell subsets. Together, our studies show that *Fcrls-2A-Cre* is a powerful and easy-to-employ mouse line to study microglia while distinguishing them from white-blood cells. This new mouse line is immediately publicly available from JAX (Stock #036591).

*Fcrls* is one of the most highly expressed genes in microglia (Hammond et al., 2019), but its function remains unknown, since mice lacking *Fcrls* appear to be normal (Oleg Butovsky, unpublished data). To minimize potential knock-out-related issues, we chose a bicistronic knock-in approach using 2A peptide preserving endogenous expression and function of *Fcrls* (Fig. 1*B*). Besides insertion of the exogenous *2A-Cre* sequence, development of the mice further required insertion of a point mutation at the PAM site of one of the CRISPR guide RNAs to prevent cutting of the targeting vector or targeted allele (Fig. 1*C*). While the functional consequence of changing a single nucleotide in the 3′UTR is unknown and difficult to predict, the lack of conservation in rats, rabbits, and humans suggests that the change might be comparatively inconsequential. Supporting this notion, qPCR analysis showed that *Fcrls* mRNA expression was unaffected (Fig. 1*G*). Moreover, *Fcrls-2A-Cre* mice were healthy, did not display any gross abnormalities, and have been bred to homozygosity (data not shown). Additionally, Cre expression in these mice did not perturb microglia phenotypes during early development (Fig. 1*G–K*), a concern which has recently been reported for another microglia CreERT2 line (Sahasrabuddhe and Ghosh, 2022).

Our studies using *Ai14* reporter mice display that *Fcrls-2A-Cre* mice recombine floxed alleles efficiently and with high fidelity (Fig. 2). The high completeness observed in microglia aligns well with *Fcrls* expression based on scRNA sequencing and in situ hybridization studies (Hammond et al., 2019; Li et al., 2019). Also, in line with these sequencing studies, we observed recombination in a substantial fraction of BAMs (Fig. 2*G–J*,*T*). Some, albeit generally much sparser, activity in some of these BAM subsets has been reported and can be inferred for the currently most specific inducible Cre lines *Tmem119-CreERT2*, *P2ry12-CreERT2*, and *Hexb-CreERT2* based on experimental data and endogenous expression pattern (Kaiser and Feng, 2019; Masuda et al., 2020; McKinsey et al., 2020). The continued difficulty to entirely avoid recombination in all BAM populations when using a highly potent Cre driver highlights a potential trade-off between completeness of recombination in microglia and distinction from BAMs. Researchers might find the inducible CreERT2 lines most suitable when specificity for microglia is the chief concern, whereas they might opt for constitutive Cre lines when completeness of recombination is the priority, particularly in the context of harder-to-recombine alleles. Yet another approach would be a successive use of Cre and CreERT2 in initial discovery and subsequent validation studies, respectively.

In this study, we evaluated Cre activity across four different Cre reporters and showed highly complete and specific combination in microglia, regardless of the reporter allele used (Fig. 3*A–K*). This is of great significance given concerns for the efficiency of some CreERT2 systems, with several reports showing lower recombination efficiencies of harder-to-recombine alleles ranging from 40 to 70%, rarely reaching >90% even for the most potent CreERT2 lines that can come with a leakiness liability (Stifter and Greter, 2020; Bedolla et al., 2023; Faust et al., 2023). The *Fcrls-2A-Cre* line recombined the reporter allele in 98% of microglia in the less sensitive R26-YFP reporter (Fig. 3*K*), which indicates extremely robust activity. Ordinarily, lower activities may be perfectly acceptable for the study of reporter-labeled cells or genetic rescue by employing a STOP-flox rescue allele. However, in a common scenario with the goal of assessing a potential loss-of-function phenotype through knock-out of two, potentially harder-to-recombine endogenous alleles rather than through activation of an exogenous reporter, maximizing recombination efficiency is essential. This is a particular concern when testing for complex phenotypic changes that might require a near complete population-level knock-out to precipitate the phenotype. *Fcrls-2A-Cre* mice should be a highly suitable tool for such applications.

With respect to temporal activity of Cre in *Fcrls-2A-Cre* mice, our studies at embryonic day 12.5 revealed recombinase activity at or even before this stage (Fig. 3*L–M*), which renders this line suitable for studies during early development. To our knowledge, similar activity has not yet been shown for the *Cx3cr1-Cre^M^* line and should not be assumed in absence of confirmation studies given available scRNA sequencing data that shows less consistent expression of this gene across several microglia subsets during development (Hammond et al., 2019; Li et al., 2019). Another line that does appear to recombine in these cells has recently been reported, named *Crybb1-Cre* (Brioschi et al., 2023). However, this line was not publicly available at the time of this study and has hence not been directly examined here.

Our examination of Cre activity in peripheral organs revealed that floxed alleles are recombined in a fraction of tissue-resident macrophages in the liver and spleen (Fig. 4*A–G*). We observed recombination in a minority, yet non-negligible fraction, of these macrophages which was much lower than that seen in *Cx3cr1-Cre^M^* mice. While recombination in even a subset of tissue-resident macrophages may appear surprising given the absence of *Fcrls* expression in adult peripheral macrophages (ImmGen.org), it is plausible based on transient *Fcrls* expression seen in some tissues at low to moderate levels during development (Mass et al., 2016). In such tissues with macrophages that turn over slowly, even low expression of Cre is sufficient for all-or-none recombination events. At this stage, we are unable to conclude if the labeled cells comprise a fraction of a general population or a nearly completely labeled specific subset of *Fcrls*-expressing macrophages. All in all, these data suggest a favorable recombination profile of *Fcrls-2A-Cre* over *Cx3cr1-Cre^M^* in peripheral tissue-resident macrophage subsets, making it a suitable line when recombination in a minority of cells is acceptable and unlikely to produce a significantly confounding phenotype.

Finally, and most significantly, our flow cytometric studies show that Cre is virtually inactive in white blood cells (Fig. 4*H–S*). This is of tremendous importance in the context of disease models since infiltration of monocytes is common in CNS diseases (Henderson et al., 2009; Ritzel et al., 2015; Shang et al., 2016). A frequently used work-around with CreER lines is to induce recombination with tamoxifen and wait for monocyte turnover, which is generally suitable for lineage tracing. However, the approach leaves ambiguity in disease contexts where monocytes with recombined alleles may at least partially drive pathology before getting turned over. Compared with other lines, *Fcrls-2A-Cre* mice offer unique advantages. The currently best publicly available constitutively active Cre line *Cx3cr1-Cre^M^* labels a large fraction of monocytes and a small fraction of lymphocytes and granulocytes (Fig. 4*P–S*) and other lines that achieve similar specificity, namely, *P2ry12-CreERT2* and *Tmem119-CreERT2*, require tamoxifen administration and are likely less efficient at recombining the allele in microglia (Kaiser and Feng, 2019; McKinsey et al., 2020). Taken together, our observation that *Fcrls-2A-Cre* mice spare monocytes and other white blood cells makes this novel line extraordinarily suitable for studies where constitutive activity of Cre is desired and where monocytes would be a major confounding factor.

*Fcrls-2A-Cre* mice present an important addition to the microglia toolbox. Compared with other mouse lines available to researchers, the major limitation of the *Fcrls-2A-Cre* line is the expression in BAMs, which can be minimized when using *Tmem119-2A-CreERT2* or *P2ry12-CreER lines* (Kaiser and Feng, 2019; McKinsey et al., 2020). Further, while the present study examined Cre activity in ectopic cell populations and potential Cre-related perturbation of microglia homeostasis, the experiments were limited in terms of developmental time points, additional cell types, and brain regions. Therefore, as for any study using transgenic animals, it will be critical for investigators to run appropriate control experiments with Cre-only control animals in the specific context of their work. In the future, more and even better constitutively active Cre lines may be developed. Most recently, *Cx3cr1* and *Sall1* loci were harnessed to create a split Cre mouse line that displayed impressive fidelity (Kim et al., 2021). At the same time, an obstacle to implementing this line for knock-out studies is its multiallelic design and relative inefficiency that may require breeding of two split-Cre loci to homozygosity in addition to the homozygous floxed allele. Perhaps, another combination for a split Cre approach achieving similar specificity while retaining high efficiency could involve targeting *P2ry12* and *Fcrls* on the same allele on chromosome 3. This would facilitate breeding and genetic crosses of the mice. Moving forward, combining these improved mouse lines with emerging strategies to deliver genetic material to microglia through AAV and other means (Lin et al., 2022; Young et al., 2023) will facilitate major advances in research into microglia function in health and disease.

## Synthesis

Reviewing Editor: Christie Fowler, University of California Irvine Decisions are customarily a result of the Reviewing Editor and the peer reviewers coming together and discussing their recommendations until a consensus is reached. When revisions are invited, a fact-based synthesis statement explaining their decision and outlining what is needed to prepare a revision will be listed below. The following reviewer(s) agreed to reveal their identity: Cassandra Gipson, Mary (Molly) Heyer.

This manuscript has been reviewed by two experts in the field. Both felt that the newly generated Cre line will be of great benefit to the research community. The data detail the generation and characterization of a novel constitutively active Cre-expressing mouse line (Fcrls-2A-Cre) targeting microglia with improved completeness and specificity. The currently available constitutive or inducible Cre lines have been notorious for leaky Cre-dependent recombination in many non-microglial cell types (poor specificity) and/or low efficiency of recombination in microglia (poor completeness). The Fcrls-2A-Cre line shows improvement in both these aspects and will be highly important for the advancement of microglial research in brain development and adult functions.

However, in the review discussion, both reviewers agreed that the manuscript would benefit from a more rigorous characterization of potential Cre activity in neurons (or lack thereof), especially as this has been such a problem in other lines. Additional comments are below:

Reviewer 1

This set of studies evaluates targeting the Fcrls gene to achieve a highly specific tool via the development of Fcrls-2A-Cre mice for the field to evaluate microglia, the resident brain immune cell that has been studied heavily for their role in various neurobiological functions. Other models such as CX3CR1-cre mouse lines have shown some issues with specificity of the cre transgene to microglia, with some reports of leakage into neurons. There are other issues to consider such as the requisite inclusion of tamoxifen into the experimental design using established mouse and rat lines, which can be a potential confounding issue depending on the outcome measures of interest. Thus, new tools that demonstrate enhanced specificity to microglia are needed, timely, and fill a gap in the rigor of prior research. The manuscript includes some critical experiments which evaluate microglia expressing the transgene during early embryonic development, which is important because microglia migrate into the embryonic brain parenchyma and thus evaluating how the transgene may modify this critical neurodevelopmental migration is an important validation of the model the authors are trying to establish. I also appreciate that the authors have made this mouse line immediately available, which is commendable resource sharing. Although the current set of studies are valuable in that they attempt to fill this critical gap and establish a new animal line, there are some existing concerns which are listed below for the authors to consider.

1. While it is exciting that the current manuscript demonstrates that the Fcrls-2A-Cre knock-in mouse line expresses Cre recombinase, there was no mention in the prior single cell RNA sequencing studies (referenced articles in the introduction include Hammond et al., 2019 and Li et al., 2019 in the introduction) that this gene does not express in neurons. This is one of the main obstacles that the field has in terms of studying microglia-specific consequences of experimental manipulations across a wide range of fields of research. In fact, studies that have claimed high impact novel models that allow for microglia manipulations such as novel viral vectors that infect microglia have also shown expression in neurons (e.g., see a supplemental figure in the high impact publication Lin et al., 2022, Nat Methods). Thus, it is important to establish novel microglia targeting models do not also impact neurons. The introduction claims, "Fcrls is not expressed in monocytes and other peripheral immune cell subsets", but there are no mentions of neuronal expression which should be included in the introduction.

2. To the point above, I appreciate that the authors tested if their model induces expression in neurons. While Figure 2L demonstrates no co-localization of tdTomato in NEUN-positive cells, this was only tested in one brain region. Further, while NeuN is detected in a large majority of neurons, it is not detected in all neuronal subtypes (e.g., see Kumar and Buckmaster, 2007). This is important because the authors state that the inclusion of the NeuN validation was meant "to rule out any expression in non-myeloid cell types". I disagree that these few validations, while sufficient to rule out expression in NeuN-expressing neurons, rules out definitively expression in all non-myeloid cell types.

3. One other issue is that while SB100 (for astroglia) and NeuN (for neurons) were utilized to evaluate leakage of Cre into other cell types, there were no studies included to evaluate if the Fcrls-2A-Cre knock-in alters astrocytic immune responses. SB100 is actually used as a marker of neural disorders and activates an inflammatory autocrine loop in astrocytes. The marker was used in the present set of studies but astrocytic function that may be impacted by the knock-in in microglia was not assessed here. Thus, although microglia homeostasis was evaluated, there may be other unintended consequences of this model that are as of yet undetermined. This is probably an important limitation that should be addressed.

4. To the point above, there are no limitations to the current studies discussed in the manuscript discussion section. It is improbable that there are no limitations here, and in fact a few have been identified by this reviewer. This needs to be included so that the field can be fully educated on the pros and cons of this new model, which may have important implications depending on the experimental questions and field of research of individuals who may plan to use this model in their studies.

5. There is no methods section that I could find detailing the immunohistochemistry of NeuN, etc. Please include these details.

6. There do not appear to be figure legends. Please include them.

7. I appreciate the rigor with which the authors tested how insertion of Cre could possibly alter microglia homeostasis. This was tested via RT-qPCR for homeostatic genes such as CD11b and CD45. However, as noted in Paolicelli et al., 2022, microglia coexist in multiple states, with transcriptomic, epigenetic, morphological, metabolomic, and proteomic changes occurring within these complex immune cells. Without another outcome measure, it does not seem like a strong argument to definitively conclude that these cells are not perturbed in early development by the insertion of Cre. Given that one of the stated main goals of the generation of this animal model is for early developmental studies to utilize for evaluations of microglia which are not impacted by the transgenic model itself, another endpoint showing evidence of a lack of baseline perturbations of microglia is warranted.

8. This statement raises a concern, "Microglia-specific genes are not expressed uniformly and some genes express at lower levels or in select microglia subpopulation" because microglia can shift expression of genes depending on environmental insult or injury and this can correlate with reactivity of microglia to environmental stimuli. The question is, does the target gene Fcrls differentially express when microglia are responding to injurious stimuli? One paper found that this gene was significantly downregulated under inflammatory conditions (Pettas et al., 2022). Can the authors speak to this in their model? It seems that additional validation of this model under induced inflammatory conditions may be warranted here to determine if this is an issue with expression of the cre transgene.

Reviewer 2

The authors describe the elegant generation and characterization of a very valuable new constitutive Cre mouse line (Fcrls-2A-Cre) targeting microglia with greatly improved specificity and completeness over current available lines such as Cx3cr1-Cre, and without the need for tamoxifen administration as is required for inducible CreER lines (which can also suffer from low efficiency of recombination). Moreover, the authors show that Fcrls-2A-Cre mice express Cre at developmental stages as early as E12.5, allowing for improved exploration of microglia throughout development. The authors describe a clear rationale for the selection of the Fcrls gene due to its specific expression in microglia throughout development, determined by mining previous single cell RNA sequencing data sets. Their knockin targeting strategy is clearly outlined and the use of a 2A site ensures separation of Cre from endogenous Fcrls protein.

The authors find that only one type of macrophage has very noticeably detectable Cre activity: border associated macrophages, BAMs, in brain; importantly, there is very low detection of Cre activity in peripheral tissue-resident macrophage populations and none to almost none in monocytes (a vast improvement over the Cx3cr1-Cre line). Critically, they do not detect Cre activity in other glial or neuronal cell types in brain. This will be highly important for the advancement of microglial research in brain development, function, and disease. This study describes thorough and rigorous characterization of a novel mouse line, is clearly written, and its results are easily compared to findings from other available microglial Cre lines; thus, it is very helpful for investigators in determining whether this line is suitable for their studies.

Specific comments

1. In Fig. 1, P8 microglia homeostasis genes appear unaffected by Cre expression, unlike previous findings for Cx3cr1-Cre, which suggests that Cre expression in the Fcrls-2A-Cre line is not toxic to microglia. It may be helpful to determine whether this is still the case in the adult.

2. Fig. 2 shows effective recombination in microglia but not astrocytes, oligodendrocytes, or neurons in brain. Perhaps I missed this, but I can't see the age of these mice in the results section or figure legend. I presume these are adult mice but this should be clarified, especially as these data immediately follow data from P8 mice in Fig. 1.

3. Fig. 2Q-S show a nice quantitative comparison of reporter expression in FACS-sorted microglia in their line vs Cx3cr1-Cre. Perhaps it would strengthen the paper to include a similar Cre driver comparison for the immunofluorescence data in brain. Researchers would be curious to compare Cre-dependent tdTomato expression across brain cell types in both lines, especially given the often leaky expression of Cre reporters in the Cx3cr1-Cre line.

4. Fig 3 D-F: please state the brain region in these panels in the results section and figure legend.

5. In the Methods section, please write out abbreviations in full when first used (e.g. LHA, RHA) and then abbreviations after.

6. There are some minor typos throughout.

7. I would suggest that the authors highlight the lack of neuronal Cre activity in their line (possibly in the introduction and/or discussion); developmental Cre activity and reporter recombination in neurons in the Cx3cr1-Cre line has been a problem that is not apparent in their new Fcrls-2A-Cre line.
